# Anti-Tumor Immunity in Solid-Organ Transplant Recipients

**DOI:** 10.3390/cancers18081216

**Published:** 2026-04-11

**Authors:** Jeffrey Sum Lung Wong, Karen Hoi Lam Li, Bryan Li, Roland Leung, Desmond Yap, Albert Chan, Tan-To Cheung, Thomas Yau

**Affiliations:** 1 Department of Medicine, Queen Mary Hospital, The University of Hong Kong, Hong Kong, China; 2 Centre of Cancer Medicine, The University of Hong Kong, Hong Kong, China; 3 Department of Surgery, Queen Mary Hospital, The University of Hong Kong, Hong Kong, China

**Keywords:** transplant oncology, anti-tumor immunity, immune checkpoint inhibitors, solid-organ tumors

## Abstract

Solid-organ transplant (SOT) recipients have an increased risk of malignancies and poor oncological prognosis compared to the general population. A central reason for both is that various factors unique to transplantation coalesce to dampen anti-tumor immunity. These include graft or immunosuppressive therapy-related T-cell dysfunction, microenvironmental changes in grafts due to ischemic/reperfusion injuries peri-transplant and comorbidities such as metabolic syndrome. Both innate and adaptive immunity are heavily implicated in cytotoxicity effected by systemic therapeutic agents, not just immune checkpoint inhibitors (ICIs) but also conventional chemotherapy and targeted therapies. Hence, impaired anti-tumor immunity may also affect the treatment efficacy of these agents. Generally, clinical data for systemic therapies in transplant recipients is constrained to retrospective and heterogenous case reports and series only, with a low level of evidence and significant risk of bias. For ICIs, the efficacy in SOT recipients is relatively well preserved in cutaneous squamous cell carcinomas but seems diminished in other tumor types compared to non-transplant recipients. Data for other agents are limited, but the efficacies of chemotherapy in SOT recipients with colorectal cancer and sorafenib/lenvatinib in LT recipients with recurrent hepatocellular carcinoma seem preserved. Given the prevailing trend of broadening the use of transplantation in patients with cancer, further clinical and translational studies to develop strategies to enhance anti-tumor immunity while ensuring graft preservation are urgently needed.

## Simple Summary

Patients who have received an organ transplant are more likely to have cancer and experience worse outcomes if they do. This is because the ability of the immune system to fight cancer, or anti-tumor immunity, is weakened by the process of transplantation, the transplanted organ itself and the medications required to prevent transplant rejection. Medications to kill cancer cells, including chemotherapy, targeted therapies and immunotherapies, all depend on the immune system for their function as well, and hence their effectiveness may be weakened in transplant recipients. Given that more cancer patients are receiving transplants, it is most important to be aware of these issues and develop further studies to improve anti-tumor immunity and thus the outcomes for these patients.

## 1. Introduction

De novo or recurrent cancer is one of the top causes of death for recipients of solid-organ transplant (SOT), driven by both increased incidence and dismal outcomes of malignancies [1]. The incidence of malignancies in transplant recipients is around two-to-three fold over the age- and sex-matched general population [2]. Conceptually, this increase is divided into two categories [2,3]. The first consists of tumors which are specific to individual transplanted organs, with examples including hepatocellular carcinomas (HCCs), native kidney renal cell carcinomas (RCCs), and lung cancers in liver, kidney and lung transplant recipients, respectively. These tumors reflect shared disease processes causing both organ failure necessitating transplantation and oncogenesis, microenvironmental changes in transplanted organs, and tumor recurrence in patients whose primary transplant indication is HCC [4,5,6]. The second category consists of malignancies whose incidences are generally elevated across all transplant types, which points towards a systemic rather than local cause [2,3]. Intriguingly, the pattern of this category provides a strong hint of impaired systemic anti-tumor immunity: tumors driven by persistent external oncogenic pressure and classically most dependent on immunosurveillance to be eradicated are nearly all more common in SOT recipients compared to the general population. These include firstly chronic viral infection-driven tumors, namely Epstein–Barr virus (EBV) related PTLD, human papillomavirus (HPV) related anogenital and oropharyngeal cancers, human herpesvirus-8 (HHV-8)-related Kaposi sarcoma and Merkel cell polyomavirus related Merkel cell carcinoma; and secondly, ultraviolet (UV) radiation-related tumors, namely cutaneous squamous cell carcinoma (cSCC), melanoma and basal cell carcinoma [2]. Correspondingly, the tumor mutation burden, which reflects the immunogenicity of the tumor, is positively correlated with the incidence rate of said cancers in SOT recipients [3]. Meanwhile, the outcomes of solid-organ malignancies in SOT recipients, as reported by multiple large-scale registries, are considerably poorer compared to general oncology patients even accounting for stage and disease [7,8,9]. Thus, underpinning these observations are two major factors: immune cell dysfunction and unfavorable microenvironmental changes, both of which promote oncogenesis and limit systemic therapy efficacy.

This review seeks to summarize and discuss these important issues, which have acquired special topicality and salience recently given the growing prominence of immune checkpoint inhibitors (ICIs) in the treatment of numerous tumors, as well as efforts to expand transplant indications to oncological conditions such as liver transplant (LT) for patients with colorectal liver metastases (CRCLMs) [10]. We focus on non-hemic malignancies, and particularly on the efficacy instead of mere safety of systemic therapeutic agents, discussing and linking both preclinical and clinical aspects of anti-tumor immunity in SOT recipients. This choice is informed by our observation that much of the current literature is primarily concerned with safety instead of efficacy, while we believe the latter to be equally deserving of discussion. Indeed, as we will show, they are often merely two sides of the same immunological coin.

Lastly, this review is narrative in nature. Most relevant clinical data is from case reports, small case series and aggregated retrospective data. Furthermore, there are significant issues such as patient and treatment heterogeneity, reporting of efficacy outcomes in a tumor-, biomarker- and treatment-line-agnostic fashion, as well as overlapping cohorts. Such issues and the paucity of high-quality data thus preclude statistical pooling and systematic review.

## 2. Immune Cell Dysfunction in Transplant Recipients

### 2.1. T Cell Dysfunction: Causes and States

**Figure 1:**
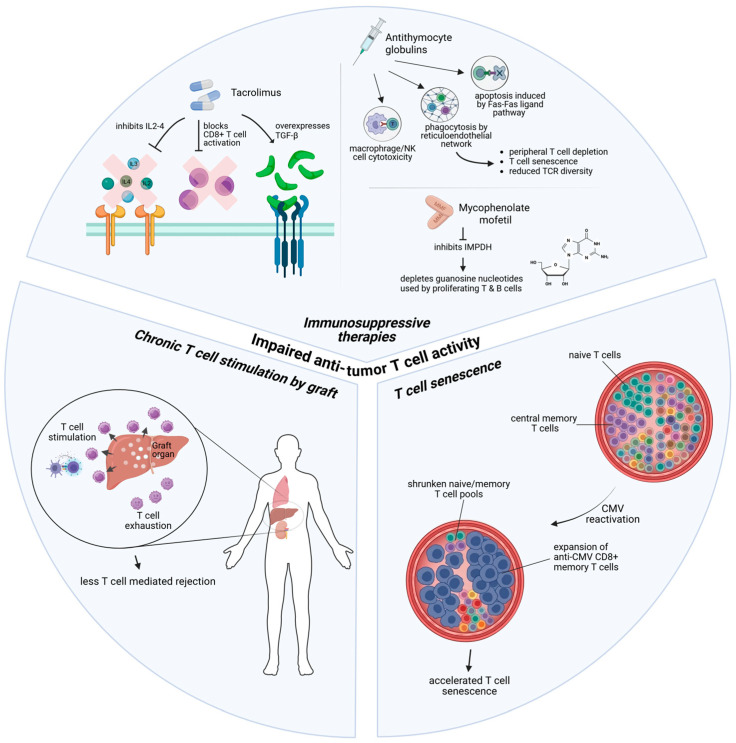
T-cell dysfunction in transplant recipients. Figure Legend: Immunosuppressive therapies, chronic stimulation by graft and cytomegalovirus infections cause T-cell depletion, inhibition of proliferation and activity, exhaustion and senescence, eventually leading to impaired anti-tumor activity. Abbreviations: interleukin (IL), transforming growth factor-beta (TGF-β), mycophenolate mofetil (MMF), inosine monophosphate dehydrogenase (IMPDH), natural killer (NK), cytomegalovirus (CMV).

Through allorecognition of graft antigens and subsequent activation, proliferation and orchestration of cytotoxic responses, T cells play a central role in mediating transplant rejections [11]. However, T cells are also the dominant effector cells of the anti-tumor immune response and immunosurveillance [12]. Hence, though the promotion of T-cell dysfunction (except for regulatory T cells) is often welcomed in SOT recipients, doing so may incur serious oncological sequelae in the long run. In general, T-cell dysfunction in SOT recipients may be the result of the following: depletion and reduced activation through the deliberate use of immunosuppressive therapies (ISTs), T-cell exhaustion due to chronic stimulation by graft, and T-cell senescence from ISTs and cytomegalovirus (CMV) infections (Figure 1).

#### 2.1.1. Immunosuppressive Therapies Related T Cell Dysfunctions

ISTs for SOT recipients are essential for graft survival and usually consist of two separate phases: the induction phase and the maintenance phase. Induction IST is administered at the time of transplantation and aims to deplete or block the activation of T cells. It is given in most patients receiving kidney or lung transplant [13,14], but its use in heart and liver transplantation remains controversial [15,16]. The two most common agents used are antithymocyte globulins (ATG) and basiliximab. ATG are polyclonal, cytotoxic immunoglobulin Gs purified from the sera of rabbits or horses against a broad spectrum of clusters of differentiation and antigens expressed on immune cells ranging from CD4+ and CD8+ T cells, to B cells and natural killer (NK) cells [17]. Fixation of these antibodies onto antigens triggers multiple cell death pathways against immune cells, including antibody-dependent cellular cytotoxicity (ADCC) by macrophages and NK cells, phagocytosis of T cells by the reticuloendothelial network, and Fas-Fas ligand pathway-induced apoptosis [17]. ATG results in near-total immediate peripheral T-cell depletion (up to 98%) [17], delayed thymic-dependent T-cell reconstitution, T-cell senescence and reduced T-cell receptor diversity [18,19,20]. Indeed, the suppressive effects of ATG on T cells may be especially prolonged compared to other immune cells such as B or NK cells [21]. Interestingly, there is also some evidence of ATG preferentially sparing regulatory T cells (Tregs), which are powerful suppressors of anti-tumor immunity [18,22]. Unlike ATG, basiliximab does not deplete lymphocytes but rather inhibits T-cell proliferation by blocking CD25, the interleukin-2 receptor (IL-2R) on activated T lymphocytes [23]. The effects of basiliximab are temporary, with saturation of circulating IL-2R epitopes lasting between 30 and 45 days after a single dose [24]. Due to its pharmacokinetics and more selective mechanism, basiliximab causes less perturbation of long-term T-cell functions compared to ATG [25]. Interestingly, in several large cohorts of kidney transplant recipients, the risk of malignancy was elevated in patients who received ATG compared to IL-2R antagonists as induction IST [26,27]. Basiliximab is used in most patients receiving a lung transplant and in patients of standard rejection risk receiving a kidney transplant [13,14]. Nevertheless, ATG is preferred in certain patients, for example in patients receiving a kidney transplant with a high risk of acute rejection, as it is associated with fewer biopsy-proven acute rejections [14,28].

Long-term maintenance ISTs are required for patients after all types of SOTs. As the risk of rejection gradually reduces with time, the intensity of maintenance ISTs decreases accordingly. Despite this, maintenance ISTs also exert major suppressive effects on T-cell functions. Contemporary maintenance ISTs usually begin with a three-drug backbone of calcineurin inhibitors (CNIs), anti-metabolites and corticosteroids [29,30], with tacrolimus and mycophenolate mofetil (MMF) being superior to other agents of the same class, respectively, and now being the standard [31,32,33]. While corticosteroids are usually tapered gradually or withdrawn entirely within months after transplantation, CNIs and MMF are often continued as part of long-term maintenance therapy and thus exert prolonged influence over the immune landscape. Both CNIs and MMF exert direct on-target suppression of major effectors of anti-tumor immunity. Calcineurin is a phosphatase with a central role in activating the nuclear factor of activated T cells (NFAT) proteins, which are in turn required for the transcriptional activation of genes for the production of lymphokines such as IL2 and IL4, T-cell differentiation and cytokine release [34,35,36]. Calcineurin is also recruited directly into the T-cell receptor signaling complex, where it reverses the inhibitory phosphorylation of the tyrosine kinase LCK, an essential step for T-cell adhesion and initiation of the adaptive immune response [37]. Thus, inhibition of calcineurin by CNI exerts a central and multi-faceted role in T-cell suppression. In addition, CNIs may be directly oncogenic by inducing overexpression of transforming growth factor (TGF)-β [38]. Meanwhile, MMF inhibits inosine-5′-monohosphate dehydrogenase (IMPDH), a key enzyme in the purine synthesis pathway, which is the only pathway available to proliferating T and B cells for the synthesis of guanosine nucleotides [39]. In addition, in T cells specifically, MMF inhibits tumor necrosis factor (TNF), interferon-gamma and IL-17 production, enhances the expression of immune checkpoints such as PD-1 and CTLA-4, and decreases the expression of co-stimulatory molecules such as CD27 [40]. MMF also exerts a range of suppressive effects on monocyte-derived dendritic cells (MDDCs), which are a key subset of antigen-presenting dendritic cells and important mediators for CD8+ T-cell activities and anti-tumor effects [41,42,43]. Thus, MMF exerts potent negative effects on nearly all facets of T-cell activity, from their co-stimulation and activation by dendritic cells to their proliferation and function.

#### 2.1.2. T Cell Exhaustion from Graft Stimulation

Chronic T-cell stimulation from prolonged antigen exposure of any source is the major driver pushing T cells into an exhausted state, which is characterized by expression of immune checkpoints, reduced proliferation and loss of effector functions [44]. In transplant recipients, the graft organ essentially acts as a huge reservoir of foreign antigens to drive T cells into exhaustion [45,46,47]. Intriguingly, this exhausted state fostered by the graft may be essential for graft survival. Preclinical models showed that transferring a set number of alloreactive CD8+ T cells to T-cell-depleted mice with liver, kidney or heart grafts resulted in an initial activation and proliferation of graft-infiltrating T cells in all mice [48]. However, liver grafts were accepted, which was correlated with liver graft-infiltrating T cells having reduced IFN-gamma and IL-2 mRNA expressions, pointing to an exhausted T-cell phenotype. In contrast, kidney and heart grafts, in which graft-infiltrating T cells showed higher effector functions and less exhaustion, were rejected [48]. Interestingly, one key driver of T-cell exhaustion seems simply to be the mass of the graft and, by extension, the antigen load. One major difference between liver grafts, which were accepted, and kidney or heart grafts, which were rejected, was that liver grafts were much larger [47,48]. Other preclinical studies showed that in mice with heart grafts, reducing the graft size by 50% leads to acute rejection instead of mere chronic transplant vasculopathy with the same amount of anti-donor T cells. Additionally, transplantation of two simultaneous kidneys, or hearts, or skin graft led to acceptance, while single grafts were rejected acutely despite a similar frequency and cytokine profile of induced anti-donor T cells [49,50].

In clinical studies, serial peripheral blood mononuclear cell (PBMC) profiling of patients with a kidney transplant showed that the percentage of exhausted CD4+ and CD8+ T cells, characterized by expression of immune checkpoints such as PD1, TIGHT and TIM3, progressively increases from month 0 to month 6 [45]. Upon isolation, these exhausted T cells were found to have diminished ATP and cytokine production [45]. An exhausted T-cell phenotype was beneficial for graft function, with the percentage of exhausted T cells correlating inversely with T-cell function and allograft interstitial fibrosis, and the increase in such T cells directly correlating with better allograft function [45,46]. Tellingly, T-cell-mediated rejection (TCMR) classically occurs in the first year of transplantation, progressively declining in frequency and activity afterwards [51,52]. This temporality reflects both progressive T-cell dysfunction and its attendant benefits [51]. However, in the context of anti-tumor immunity, exhausted effector T cells are unable to mount a sufficient cytotoxic response to kill cancer cells and in fact represent a hallmark of tumor immune escape [53,54]. Although microenvironmental differences between various drivers (e.g., chronic infection versus malignancy) result in distinct molecular mechanisms of exhaustion [55], evidence from studies of patients with chronic viral infections suggested that regardless of cause, exhaustion of circulating T cells is associated with increased cancer risk and progression [56,57,58]. In addition, specific to a liver transplant for patients with HCC, exhausted graft-infiltrating T cells, analogous to the microenvironment created by chronic hepatitis, may impact local anti-tumor immunity against any intrahepatic tumor recurrence.

#### 2.1.3. Accelerated T Cell Senescence

Lastly, accelerated T-cell senescence is observed in SOT recipients [20,59]. Although both T-cell senescence and exhaustion cause functional impotence, they differ in that there is increased DNA damage, reduced telomere length and telomerase activity, and irreversible cell cycle arrest in the former but not the latter [60,61]. Consequently, functional reinvigoration through immune checkpoint blockade is possible for exhausted T cells but not for senescent ones [60]. T-cell senescence can be induced by ISTs such as ATG (as discussed above) and chronic tacrolimus exposure, which is associated with increased senescent CD8+ CD57+ T cells and less-responsive T-cell receptor pathways [62]. Another major driver is CMV infection. CMV usually behaves as a chronic, latent viral infection which sporadically reactivates [63]. These episodes of reactivation stimulate the expansion of CD8+ memory T cells against a variety of viral antigens and virus-encoded immunomodulators [63,64,65,66]. Of note, these T cells are characterized by various hallmarks of senescence [65]. CMV appears to be highly immunogenic, and infection causes major restructuring of lymphoid subsets. A large portion (around 10% in healthy carriers and up to 40% in the elderly) of circulating T cells become dedicated to anti-CMV response, accompanied by significant reductions in naïve CD8+, CD4+ and central memory T-cell pools [64,66]. This phenomenon, known as ‘memory inflation’, is associated with accelerated senescence of T cells, as the prominence of anti-CMV T cells skews the global T-cell pool [63,65]. The above process is accelerated in SOT recipients who are at a high risk of CMV reactivation due to immunosuppression. Indeed, studies conducted in liver, kidney, lung and heart transplant recipients have all shown that prior CMV infection or acute episodes of CMV reactivation are associated with increased T-cell senescence, which may be associated with less graft rejection [59,65,67,68]. Although CMV seropositivity by itself is not associated with increased cancer risk in SOT recipients [69], several lines of evidence suggest deleterious effects of T-cell senescence on anti-tumor immunity. In liver transplant recipients, T- and B-cell senescence may be associated with increased risks of cancer [70]. Circulating senescent CD8+ and CD4+ T cells are associated with reduced benefit to ICIs in patients with advanced non-small cell lung cancer (NSCLC) and metastatic melanoma, and poorer prognosis in patients with advanced gastric cancer [71,72,73]. The loss of expression of the co-stimulatory molecule CD28 on circulating CD8+ T cells, which is commonly seen with aging and is one of the markers of senescence, is also associated with adverse prognosis in patients with advanced NSCLC and metastatic breast cancer receiving chemotherapy [74,75,76,77].

### 2.2. Other Immune Cells

Aside from T cells, other immune cells are also affected by SOT. As mentioned above, ATG and MMF suppress lymphocytes non-selectively and thus also induce B-cell depletion [21,78]. The anti-CD20 antibody rituximab, which profoundly depletes B cells, is the mainstay in ABO-incompatible transplants and in the treatment of antibody-mediated rejection (ABMR) [79,80,81]. Though less characterized and therapeutically targeted than T cells, B cells have increasingly been recognized to play an role in anti-tumor immunity [82]. Tumor-infiltrating B cells serve as professional antigen-presenting cells (APCs) to stimulate anti-tumor T-cell responses [83,84,85]. In addition, antibodies secreted by B cells also trigger cytotoxicity against tumor cells by antibody-dependent phagocytosis and cellular cytotoxicity, and IgG binding to tumor cells is associated with higher response to ICIs in patients with RCC [86,87]. Thus, ISTs used in SOTs may also cause profound B-cell suppression and have deleterious effects on anti-tumor immunity.

The innate immune system may also be affected in SOT recipients. MMF and ATG again appear to inhibit the proliferation and activities of NK cells and dendritic cells [17,88,89,90]. CNIs also appear to have an important effect by mechanisms such as inhibiting toll-like receptor-dependent activation of monocytes/macrophages and NK cells [91,92]. Interestingly, CNIs are also associated with expansion of the highly immunosuppressive myeloid-derived suppressor cells (MDSCs), both in preclinical models and in clinical studies involving patients receiving kidney, lung or corneal grafts [93,94,95,96]. The roles of the innate immune pathway in tumor response are complex and have been reviewed in detail elsewhere [97]. Briefly, dendritic cells are crucial APCs to enable T-cell activation, whilst NK cells and certain subtypes of macrophages (e.g., M1) have direct anti-tumor cytotoxicity. Thus, important enablers and effectors of the overall immune response may be suppressed by ISTs. The expansion of MDSCs may also be associated with profound suppression of anti-tumor activity. MDSCs inhibits T-cell activity in a myriad of ways including oxidative stress via reactive oxygen species generation and secretion of various anti-inflammatory cytokines such as TGF-β and IL-10 [98]. Crosstalk between MDSCs and T cells, B cells, NK cells and dendritic cells inhibits maturation and differentiation, proliferation, trafficking and the immune response of all these immune cells and significantly dampens anti-tumor responses [99]. In fact, high levels of circulating MDSCs are strongly associated with tumor metastasis, poor prognosis, and suboptimal response to anti-cancer therapies in various malignancies [100,101].

## 3. Microenvironmental Changes

**Figure 2:**
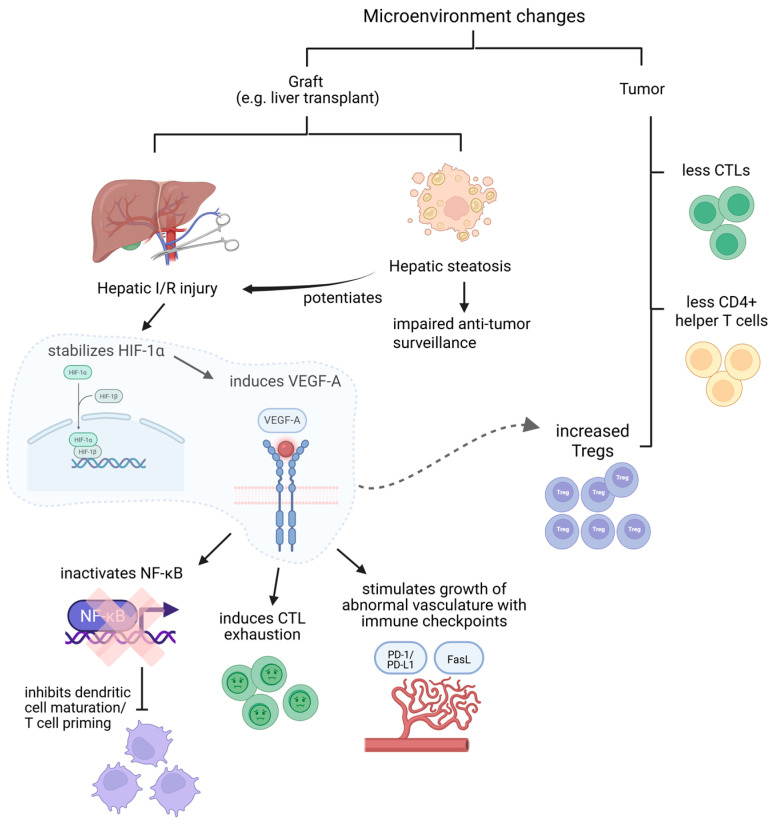
Microenvironmental changes impacting anti-tumor immunity. Figure Legend: In transplant recipients, graft microenvironmental changes such as ischemic/reperfusion injuries and hepatic steatosis in liver grafts cause immunosuppression by various mechanisms and impair anti-tumor immunity. The tumor microenvironment is also skewed towards immunosuppression. Abbreviations: cytotoxic T lymphocytes (CTL), ischemic/reperfusion (I/R), hypoxia-inducible factor 1α (HIF-1α), NF-kB (NF-kappa B), vascular endothelial growth factor (VEGF), regulatory T cells (Tregs).

Anti-tumor immunity is also heavily dependent on microenvironmental factors both in the organ and in the tumor (Figure 2). The tumor microenvironment (TME) is skewed towards immunosuppression in transplant recipients [102]. cSCCs are one of the classical and most prevalent post-transplant SOTs. Compared to immunocompetent patients with cSCCs, the TME of transplant recipients with cSCCs is characterized by reduced infiltration of important effectors of anti-tumor immunity such as CTLs, CD4+ T helper cells and antigen-presenting dendritic cells, as well as increased level of Tregs [103,104]. Similarly, reduced CTL cytotoxicity and antigen presentation as well as increased Treg inhibition have also been described in renal transplant recipients with CRCs [105]. The density of tertiary lymphoid structures, which is associated with improved response to immunotherapy [106], has also been found to be lower in tumor specimens from SOT recipients [102].

Graft microenvironmental changes may also have a significant impact on anti-tumor immunity. These are most pertinent for liver transplant (LT) patients, as they are most at risk of having tumors affecting the graft directly. Intraoperatively, the liver graft unavoidably sustains ischemic and reperfusion injury during warm and cold ischemic times. There is strong evidence that prolonged ischemic times are associated with a significant increase in the risk of HCC recurrence through multiple mechanisms [107]. The absolute difference in tumor relapse risk persists and even increases years after transplantation [108,109], suggesting that acute phase insults have long-term sequelae towards the establishment of a pro-tumorigenic microenvironment. Focusing on anti-tumor immunity, hypoxia due to ischemia stabilizes hypoxia-inducible factor 1α (HIF-1α), which activates protective pathways to allow for the survival of normal cells [110]. However, HIF-1α also induces vascular endothelial growth factor (VEGF), which suppresses anti-tumor immunity through inactivation of NF-kappa B signaling to inhibit dendritic cell maturation and T-cell priming [111], inducing cytotoxic T-cell exhaustion through upregulating TOX-mediated transcriptional reprogramming [112], and stimulating growth of abnormal vasculature which expresses immune checkpoints such as PD-L1 and FasL [113]. Both HIF-1a and VEGF also cause accumulation of Tregs [114,115]. Elevated Tregs have been shown to be associated with HCC recurrence after transplantation [116]. Indeed, Tregs probably have a major role towards transforming acute phase insults to long-term, late-phase tumor recurrence [116]. Increased circulating Tregs and intra-graft CXCL10, which mobilizes Tregs, have been found in patients who received small-for-size living donor grafts at risk of acute graft injury post-transplant [116]. In mouse models, both direct depletion of Tregs and knockout of CXCL10, which reduced mobilization and recruitment of Tregs, inhibited late-phase tumor recurrence after ischemic/reperfusion injuries [116].

Another important microenvironmental factor is hepatic steatosis, which LT recipients frequently suffer from. Steatosis can pre-exist in the liver graft, with up to 30–50% of donors in the West having hepatic steatosis [117,118], or occur de novo post-transplant (in 25–30% of recipients) [119]. The latter is driven by the high incidence of metabolic syndrome in post-LT recipients (partly due to the side effects of CNIs) [120]. Pre-existing graft steatosis potentiates ischemic/reperfusion injuries due to disrupted microcirculations [121]. In addition, in a series of landmark preclinical studies, steatosis is associated with auto-aggressive T cells against hepatocytes, impaired anti-tumor surveillance and lack of response to anti-PD1 [122,123]. All such microenvironmental changes may have profound and prolonged effects on anti-tumor immunity, increasing risk of tumor recurrence and impairing treatment efficacy by systemic agents.

## 4. Efficacy of Systemic Anti-Cancer Therapy in Transplant Recipients

### 4.1. Immune Checkpoint Inhibitors (ICIs)

ICIs targeting the programmed death-1 (PD-1) and cytotoxic T-lymphocyte associated protein 4 (CTLA-4) pathways have transformed cancer care in the past 10 years. Through stimulation of T cells in the initial activation and priming phase by blocking CTLA-4, and reinvigoration of exhausted T cells in the effector phase by blocking PD-1, significant prolongation of survival in patients with advanced cancer and even long-term remission in a minority of patients have been achieved [124]. This comes at a risk of increased autoimmune side effects and, in transplant recipients, allograft rejection. Indeed, immune checkpoints and transplant rejection are deeply intertwined: one of the first known biological roles of CTLA-4 has been the promotion of transplant rejection, and blockade of T-cell activation by CTLA-4 Ig can prevent allograft rejection. In addition, the various dysfunctional T-cell states beneficial to graft tolerance, most notably T-cell exhaustion, are either associated with or directly mediated by the expression of immune checkpoints. These relationships highlight the potential risks involved in using ICIs in SOT recipients [125,126].

#### 4.1.1. General Considerations in Data Interpretation

Indeed, much has been written on the safety of ICIs post-transplant, even though most currently available data is retrospective in nature and the overall number of patients reported remain small. In general, the available evidence shows that the risk of acute graft rejection is between 30 and 40%, with 15–20% of patients developing graft loss [127,128,129]. To justify such significant risks of IST intensification and irreversible organ failure, the expected efficacy needs to be high. However, the reported efficacy data suffers from several important issues. Most data are from case reports or small case series, which were aggregated into systemic reviews and meta-analyses. Despite aggregation, patient populations remain small when looking at individual tumor types, especially for tumors other than skin cancers. The source data consist of very heterogenous patients even in major categories such as tumor and transplant types, ICI regimen used and line of therapy, etc. In addition, important predictive and prognostic information specific to each tumor type, such as biomarker status in melanoma and lung cancer, as well as Child–Pugh status in HCC, remain unreported, making it impossible to determine the context and thus expected efficacy of ICIs. Case series and reports are also particularly prone to publication bias, as patients with ideal outcomes, such as those with tumor response and without rejection, may be selectively reported. Different aggregated cohorts and meta-analyses may have highly overlapping source data and do not represent entirely independent data sets. Finally, there is a strong tendency to report efficacy endpoints in a tumor-agnostic manner (such as the ORR of the whole cohort comprising different tumor types), which is unfortunately of little utility for further analyses or as a reference for clinical care given the wide disparity in prognosis and ICI efficacy in different tumor types.

With these limitations in mind, data is most abundant for ICI use in patients with the following four types of advanced malignancies: cSCCs, melanoma, HCCs and lung cancer, which together accounts for northwards of 80% of reported ICI use in transplant recipients in contemporary cohorts [127,130].

#### 4.1.2. Anti-PD1/L1 in Transplant Recipients

ICI use in patients with SOT and cSCC has the most abundant evidence and most favorable outcomes out of all tumor types [127,129]. The largest cohort to date, reported by Saleem et al. recently, had more than 100 patients and showed an objective response rate (ORR) of 61%, progression-free survival (PFS) at 1 year of 46.7%, and overall survival (OS) at one year of 57.1% [127]. These results are comparable to phase 2 registration trials in the general population, which reported ORRs of around 40–45%, one-year PFS at 40–60% and one-year OS of 70–80% [131,132]. Interestingly, a phase 1 trial containing 12 kidney transplant recipients who were given cemiplimab for advanced cSCC also showed a promising ORR of 46%, and a median PFS and OS of 22.5 months, without any allograft rejection events [133].

The available literature paints a less promising picture for melanoma. Out of a cohort of 103 patients reported by Saleem et al., who were mostly given single-agent anti-PD1 but with line of therapy unknown, an ORR of nearly 50% was reached (48.5%). However, the PFS and OS at 1 year are only around 30% and 37.6%. Although the ORR was preserved, PFS and OS compared unfavorably to phase 3 trials of anti-PD1. In Keynote-001, pembrolizumab demonstrated a 1-year PFS 35% in the overall population and 52% in treatment-naïve patients, as well as a 1-year OS of 66% overall and 73% in treatment-naïve patients [134]. Meanwhile, single-agent nivolumab showed a PFS at 1 year of 32% and OS of 58.9% in late-line settings, and 3-year PFS of 32% and OS of 52% in first-line settings [135,136]. Real-life cohorts in general melanoma patients also showed a median OS of around 22 months and 40–50% 3-year survival [137,138,139].

Data is scarcer for HCCs and lung cancer. In LT recipients with recurrent HCCs and without rejection after ICIs, a recent systemic review which included 31 patients showed an OS of only 7 months and PFS of 2.8 months. ICIs were mainly used in the second line, while the pre-treatment liver function was not reported. The ORR was 19%, and patients who responded reported impressive long-term OS, but 67% of patients had progressive disease (PD) as their best response (BOR) [140]. In comparison, second-line single-agent anti-PD1 clinical trials reported OS of around 13–15 months [141,142], with a similar ORR but only 30–40% of patients having PD as BOR. Real-life cohorts of non-SOT patients given single-agent anti-PD1/L1 as the second line or beyond also reported a similar ORR but a median OS of around 10–11 months [143,144,145]. Finally, the results of 11 transplant recipients given ICIs for lung cancer, which was the largest cohort reported so far, was recently published [146]. The PDL1 was ≥50% in one patient and positive in three others, with five and two patients having negative and unknown PD-L1, respectively. The median PFS and OS were 4.0 and 4.6 months, respectively, while the ORR was 45.5% and 45% of patients had PD as BOR. Meanwhile, PD-L1-agnostic trials generally reported an OS of 10 and 22 months in pre-treated and first-line patients, respectively [147,148], while real-world data described OS in pre-treated patients of around 7.9 to 14.6 months and around 18–24 months in first-line patients, and ORRs between 17 and 25% in the former and around 50% in the latter [149].

#### 4.1.3. Anti-CTLA-4 in Transplant Recipients

In HCC, melanoma and MSI-high CRC, combined blockade of CTLA-4 and PD1/L1 yielded improved OS and ORR over anti-PD1/L1 alone, at a cost of increased incidence of immune-related adverse events (IRAEs) [150,151,152,153]. The unique clinical effects of combined blockade can also be seen from responses in patients who were resistant to single blockade, as previously reported by our group [154]. Anti-CTLA-4 affects the immune priming and proliferation phases of T cells, unlike anti-PD1/L1 which affects the effector phase [155]. The addition of anti-CTLA-4 has unique immunological effects including a broader clonality of T cells and memory T-cell stimulation [156,157]. Importantly in the context of transplant recipients, CTLA-4 is directly expressed on all Tregs and has essential roles in Treg function, development and survival [158]. Antagonizing CTLA-4 has been shown to deplete Tregs, and interestingly only selectively in the tumor microenvironment (TME) but not in blood or lymphoid organs, potentially because the levels of CTLA-4 expression by Tregs in the TME are much higher [155]. In contrast, administering anti-PD1/L1 only has an equivocal effect on the depletion of Tregs [155,159,160].

Reports of anti-CTLA4 use in transplant recipients are scarce. In the cohort reported by Saleem, only 35 patients (10.2%) and 19 (5.5%) received dual ICI and anti-CTLA-4 use, respectively, with patients with melanoma constituting most of these patients (75.9%) [127]. Even though the numbers are small, several observations can be made: For dual-ICI use, the incidence of graft loss at 1 year appears to be increased (HR 2.22, 95% CI 1.13–4.35), reaching 42.2% vs. 21.0% compared to single-PD1/L1 alone. Yet, although treatment specific outcomes in individual tumor types are not available, the overall incidence of cancer-related death appears to be reduced (HR 0.42, 95% CI 0.18–0.96) compared to single-agent PD1/L1. Meanwhile, anti-CTLA-4 alone does not appear to have a significantly increased incidence of rejection or graft loss and with comparable cancer-related death [127,129,161]. The rates of graft loss appear to mirror results in phase 3 trials, with grade 3–4 IRAE doubled in dual-ICI use when compared to single-agent anti-PD1 or anti-CTLA-4 alone [136]. However, and somewhat reassuringly, they do not seem to suggest that CTLA-4 blockade alone is particularly prone to triggering rejections compared to PD1/L1 blockade.

#### 4.1.4. Balancing Risks and Benefits

##### General Characteristics of ICI Response in SOT Recipients

Juxtaposing different clinical studies is an exercise rife with potential biases and confounders, and one should be mindful of this while interpreting the above data. In particular, the treatment intensity (e.g., dose and frequency of ICIs), biomarker landscape, line of therapy and performance statuses of SOT recipients, none of which has a direct bearing on anti-tumor immunity, may deviate significantly from patients in clinical trials or general real-world studies. Overall, and keeping in mind the limitations of evidence and potential confounding as discussed above, a general pattern can be observed: with the exception of cSCCs, transplant recipients may achieve a comparable ORR with ICIs compared to general oncology patients, with those who responded doing reasonably well. However, and even without rejection, fewer patients have a stabilization of disease, and PFS and OS are considerably worse in general.

This ‘all or nothing’ phenomenon strongly hints at most transplant recipients having defective anti-tumor immunity even with maximal withdrawal of ISTs upon diagnosis of malignancy. In the absence of more granular data, we can only postulate the causes of this divide between ORR and survival. Fewer patients may have been able to achieve stable disease. Additionally, patients who responded may have had a lower quality of response, characterized by less tumor shrinkage and a shorter duration of response (DOR), thus reducing the survival benefit conferred by tumor shrinkage. These possibilities should be explored in future studies. Lastly, ICIs classically induce a long ‘tail’ of response in 10–15% of patients [162]. Whether this is also observed in SOT recipients would be an important, but currently unreported, indicator of ICI effect and may hint at specific underlying immunological deficits, as DOR and long-term survival are uniquely associated with memory T-cell activation [163].

##### Patient Selection: The Higher the Risk, the Higher the Reward?

**Figure 3:**
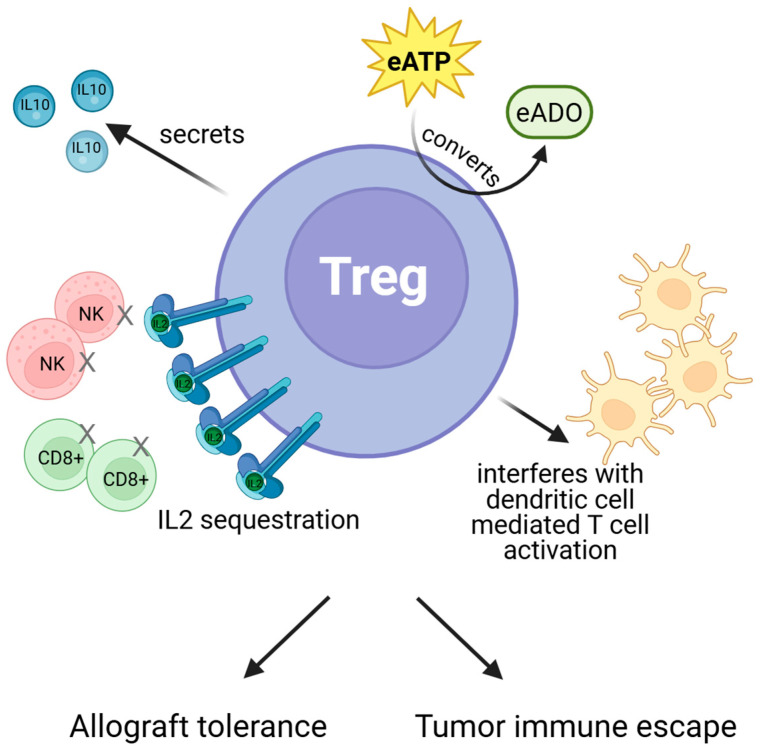
Regulatory T cells: a double-edged sword. Figure Legend: Regulatory T cells mediate both allograft tolerance and tumor immune escape against immune checkpoint inhibitors via IL2 sequestration, IL10 secretion, conversion of extracellular ATP to adenosine, and blocking T-cell activation. Abbreviations: regulatory T cells (Treg), extracellular adenosine triphosphate (eATP), extracellular adenosine (eADO), natural killer cells (NK), interleukin (IL).

Another interesting possibility is that the observed diminished efficacy of ICIs is due to patient selection: those who developed acute rejection in fact had the highest chance of benefiting from ICIs were it not for organ rejection and therapy discontinuation. As discussed above, graft tolerance and tumor immune escape share several mechanisms. By extension, this observation also suggests that factors which promote acute rejection after ICI administration may promote anti-tumor immunity as well [164]. One of the most important such factors is Tregs (Figure 3). Tregs are major contributors to allograft tolerance by a wide array of mechanisms such as secreting immunomodulatory IL10, converting pro-inflammatory extracellular adenosine triphosphate (ATP) into anti-inflammatory adenosine, and sequestering IL-2 to control expansion of CD8+ T cells and NK cells, as well as expressing CTLA-4 to interfere with dendritic cell-mediated T-cell activation [158]. Though these mechanisms are also shared by other cells, Tregs are uniquely able to deploy the full range of them, in a situation- and time-specific manner [158]. In both liver and kidney transplant recipients, those who develop acute or chronic rejection have been found to have fewer Tregs compared to those who do not [165,166]. Indeed, Treg-based products have been explored as a potential method of alternative post-transplant immunosuppression and shown to be similarly efficacious compared to conventional ISTs [167]. However, as discussed above, Tregs also negatively affect anti-tumor effects, including those of anti-PD1/L1 ICIs [155]. In trials involving ICIs in multiple tumor types, higher levels of Tregs in both the circulation and tumor microenvironment have been established as a predictive biomarker for inferior response [168,169]. Hence, transplant recipients who develop acute rejection after ICIs might in fact have an immune phenotype which predisposed them to having superior anti-tumor outcomes, and vice versa.

Available clinical data for this possibility is limited in quality and by potential biases. Runger et al. reviewed 144 patients from case reports and series, reporting a rate of tumor response while retaining the transplanted organ of 30.8% [129]. Contrary to expectations, the ORR was 1.7-fold higher in those who retained their transplant compared to those with rejection (41.2% vs. 24.2%). Several factors may explain this observation: the numbers reported were again very small (only 40 and 8 patients had a response without or with rejection, respectively) and there is a considerable risk of publication bias. Additionally and more specifically, rejection typically occurs quickly upon starting ICIs, with a median of only two doses given before rejection [127], which is before the median time-to-response for ICIs (around 2–3 months) across different tumor types even in trial settings [150,170,171]. Given also that the reported cases are all in real-life settings with less frequent radiological tumor reassessment compared to trial settings, many patients with rejection might already have discontinued ICI and been subjected to high-dose immunosuppression before any anti-tumor response could have been expected to occur or be observed. Nevertheless, it would be of tremendous interest to study the immune characteristics of the subset of patients in which the fragile balance between lack of rejection and anti-tumor immunity was maintained.

##### Differences by Transplant Type

**Figure 4:**
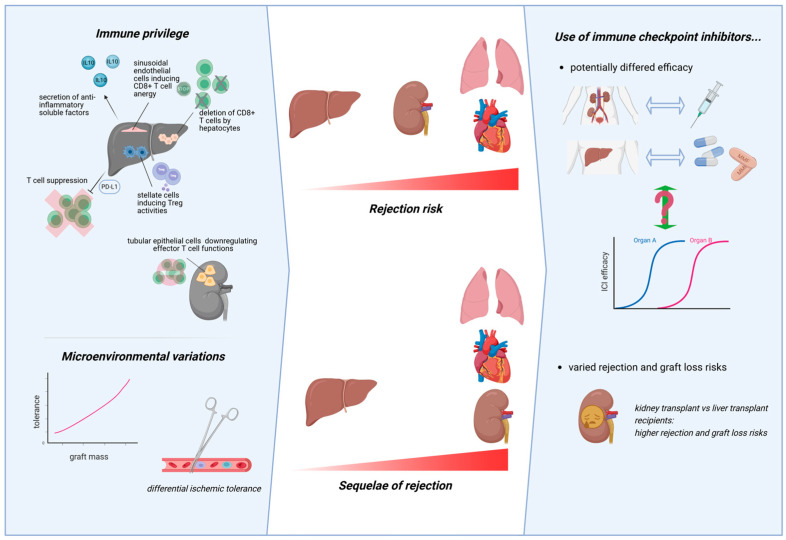
Graft organ-specific differences in ICI risks and benefits. Figure Legend: Different graft organs have different baseline immune milieu and microenvironmental variations, risk and sequelae of rejection. Liver grafts are generally the most immune-privileged. Hence, they are likely at a lower risk of rejection after ICI administration, although the efficacy of ICIs per graft organ type is unknown. Abbreviations: interleukin (IL).

Importantly, the risk–benefit ratio of ICIs likely differs by transplant type. Indeed, all transplanted organs are not created equal (Figure 4). Swine and mouse models of major histocompatibility complex-mismatched transplantation have consistently reported a hierarchy of allograft tolerance, with liver grafts being least likely to be rejected, followed by kidney, lung and lastly heart grafts [172]. In humans, direct comparison of cellular rejection risks across different organ grafts is uninformative due to differences in the ISTs used. However, the relative frequencies of operational tolerance (OT), defined as stable graft function without ISTs, also corroborate this hierarchy. OT was achieved in around 20–40% of selected patients who underwent liver transplantation [173,174,175]. In contrast, the rate of OT for kidney transplantation is generally reported to be much lower, with some estimates as low as 0.03% [176,177]. Unlike kidney transplant recipients for whom larger case series describing OT still exist, OT appears to be rarer still in lung and heart transplant recipients, with only two cases of OT for lung or heart transplantation having ever been reported [178,179,180]. Furthermore, ABMRs are rare in liver transplant recipients, while they are commonly seen in kidney, lung and heart transplant recipients with major negative impacts on graft survival [181,182,183,184,185].

Aside from different rejection risks, the sequelae of rejections also differ. Liver grafts are least affected by rejection: acute cellular rejections are seldom graft-threatening, can be entirely without clinical or biochemical graft dysfunction, and are mostly easily suppressible [186,187,188]. The robust regenerative capacity of the liver also ensures that rejection episodes may not lead to long-term graft dysfunction, although more recent data in real-world settings reported that deleterious effects are still observed [189,190]. In contrast, acute rejections of kidney and lung graft often lead to early or even rapid graft loss, as well as a high incidence of scarring and subsequent permanent graft dysfunction [191,192,193,194]. Indeed, up to 40–60% of lung transplant recipients develop Bronchiolitis Obliterans Syndrome by 5 years post-transplant, a form of chronic rejection strongly associated with graft failure and death [195,196,197]. The sequelae of rejection in heart grafts are arguably even higher still. Rejections, despite being treated, may lead to transplant vasculopathy and left ventricular dysfunction with risk of fatal ischemia or arrhythmias and are strongly associated with an increased risk of sudden cardiac death [198,199].

A detailed discussion of the factors underpinning these differences is beyond the scope of this review. Briefly, organ- specific cells may differentially downregulate alloreactivity [172]. Hepatic immune privilege is an essential adaptation due to the liver’s constant exposure to large amounts of foreign antigens from the portal circulation [200]. This is mediated by the secretion of anti-inflammatory soluble factors such as IL-10 and T-cell suppression via expression of PDL-1 by Kupffer cells, endocytosis and deletion of CD8+ T cells by hepatocytes, induction of Tregs by hepatic stellate cells, and induction of CD8+ T-cell anergy by liver sinusoidal endothelial cells [201,202,203,204,205,206]. In kidneys, renal tubular epithelial cells can also downregulate effector T-cell functions [207,208]. Microenvironmental factors also play a role. As discussed above, the mass of the graft is possibly correlated with tolerance by increasing T-cell exhaustion. The tolerance to ischemia of different organ types may also differ. Ischemic injuries to graft organs induce inflammatory responses and enhance alloreactivity but are unavoidable during transplant surgery [172,209,210]. However, the degree of ischemic injuries and proinflammatory response may be organ-specific, with the heart or lung being affected more than kidney grafts in swine models [211].

The combined clinical significance of these factors is two-fold: the use of ISTs in transplant types with a higher risk of rejection (e.g., induction ATG in certain patients with a kidney transplant) is more intensive and the attendant impact on anti-tumor response may be greater, which by extension potentially affects responses to ICIs. Unfortunately, the quality of relevant clinical data is poor. The available literature only reported ISTs at the time of ICI initiation, instead of stratifying ICI outcomes according to the intensity of ISTs or history of induction ISTs [127,212]. The difference in ICI response in individual tumor types stratified by transplanted organ is also not reported. Some tumor-agnostic data on the PFS of ICIs by transplant types are available, but these are difficult to interpret meaningfully due to the reasons discussed above [127]. Secondly, the risk of rejection and graft loss differs by transplant type after the use of ICIs. Relevant data is more abundant here, perhaps reflecting the focus on safety instead of efficacy of the current literature, with the vast majority of reports being from liver or kidney transplant recipients [127,161,212]. Kidney transplant recipients are at a higher risk of rejection, with rates of around 40% (compared to around 5% in historical cohorts) of acute rejection and the majority of which resulting in a graft loss [127,212]. As reported by Saleem et al., the risk of both rejection and graft loss appears to be lower in non-kidney transplants (HR 0.58 and 0.36, respectively), with most of these patients (around 75%) having received a liver transplant [127].

##### The Unique Potential of ICIs

Ultimately, giving ICIs to transplant recipients involves making difficult decisions regarding whether to do so and the timing and regimen used. Even though ICIs have the highest risk of triggering rejections out of all systemic therapy options, they also have three unique roles justifying their use: firstly, for nearly all malignancies, ICIs are often the only option to potentially achieve long-term disease control; secondly, ICIs may be vastly superior to other options, such as in BRAF-negative melanoma or MSI-high tumors; thirdly, as T cells may be exhausted at baseline for transplant recipients due to chronic graft stimulation and ISTs, ICIs may be uniquely able to reinvigorate anti-tumor immunity. Whether other therapies should be exhausted before ICIs are given is contentious: ICI efficacy has been well known to diminish noticeably with each additional line of preceding therapy, while the risk of grade 3–4 IRAE with the same ICIs appears to be increased for earlier-line use in melanoma and lung cancer [136,213,214] but similar for HCCs [142,150,215,216]. In tumor-agnostic cohorts of transplant recipients, later-line use of ICIs appears to be associated with fewer rejections overall [161]. Therefore, earlier use of ICIs may be a ‘high risk, high reward’ choice. The same can also be said for dual-ICI use, especially for tumor types in which dual-ICI use confers considerably more benefit compared to single-agent ICI alone. Given that the risk of irreversible organ failure is substantial whatever the ICI regimen used, and that progressive malignancy is invariably fatal in any case, clinicians may consider the early use of ICIs, dual-agent if necessary, judiciously and after a detailed discussion with transplant physicians and, most importantly, patients regarding the risks and benefits associated.

### 4.2. Other Systemic Anti-Cancer Therapies

Dependence on immune-based cytotoxicity is not exclusive to ICIs but shared by many other modalities of systemic therapy. Although conventional chemotherapies were traditionally thought to exert their anti-tumor effects mainly via DNA damage, replication arrest and other direct pathways, preclinical and clinical evidence has increasingly demonstrated that cytotoxicity is also mediated through immune responses [217]. Taxanes, doxorubicin and platinum-based agents have been shown to leverage CD8+ and CD4+ T-cell responses to kill tumor cells, enhance dendritic cell activity and deplete Tregs [218,219,220,221]. Meanwhile, cyclophosphamide and doxorubicin have been shown to deplete intratumoral M2 tumor-associated macrophages (TAMs) and promote the innate immune response against tumor cells by M1 TAMs [222]. Ample clinical evidence has shown that ICIs have synergistic effects with chemotherapy in lung and breast cancers [223,224,225]. Likewise, oncogenic signaling pathways have been shown to strongly interact with tumor immune escape. For example, loss of PTEN leads to an increase in PD-L1 protein expression via activation of the PI3K, AKT and mTOR pathways. Blocking these pathways by targeted therapies downregulates PD-L1 and potentiates anti-tumor immunity [226,227,228]. One of the most well-studied agents is trastuzumab [228], which exerts a wide variety of immune effects including cross-presentation of tumor antigens, promotes cytotoxic T-cell priming and immunogenic cell death. Clinical studies have shown that exposure to trastuzumab increases T-cell markers and PD-1 positivity [229]. In fact, there is evidence that the effect of trastuzumab may be heavily dependent on the immune system, with the degree of immune cell infiltration correlating strongly with the clinical response [229]. Adding anti-PD1 to trastuzumab also significantly enhanced the objective response rate rate in patients with HER2-positive gastric cancer [230]. Certain small-molecule inhibitors may also depend on immunogenic cell death for their anti-tumor effect. In mouse models, lenvatinib upregulated CXCL10 and CCL8 to promote tumor infiltration of T cells, induced proliferation and enhanced interferon-gamma production by cytotoxic T cells, as well as promoting natural killer (NK) cell infiltration into tumors [231,232,233]. The anti-tumor effect of lenvatinib was attenuated by the depletion of CD8+ T cells or NK cells, or when lenvatinib was used in an immunodeficient instead of immunocompetent TME [233].

Given the central role of anti-tumor immunity in chemotherapies and targeted therapies, it is of great interest to elucidate the impacts of transplantation and ISTs in transplant patients receiving these agents. Unfortunately, clinical data, apart from targeted therapies in patients with HCC post-LT, is scarce and heterogenous. Cohorts are small and heterogenous, and there is no data on many widely used anti-cancer agents or on common tumor types such as breast cancer. Much more evidence is needed to determine if the efficacies of chemotherapies or targeted therapies in SOT recipients are preserved, and if and how they are affected by SOT-related immunosuppression.

For chemotherapy, Smedman et al. reported a prospective cohort of patients who had LT for unresectable CRCLM and subsequently recurred requiring palliative chemotherapy [234]. All patients received one to five lines of pre-LT chemotherapy as well. In the initial cohort of 23 patients, 12 responded or had stable disease, and treatment was generally well tolerated. An updated analysis with 33 patients showed a median OS of 18.5 months [235]. No graft loss was reported. The unique treatment sequence and small number of patients is difficult to contextualize, but the OS of patients receiving second-line chemotherapy is generally around 13–14 months [236]. Another small case series also reported that long-term tumor control may be feasible with chemotherapy and locoregional therapy in LT recipients with metastatic CRC [237]. Young et al. published a cohort of 10 patients who received palliative chemotherapy for NSCLC post-SOT and reported an median OS of 8 months [238], while another cohort with 7 lung cancer patients reported a median OS of 6 months with chemotherapy [239]. In comparison, the median OS of unselected patients with NSCLC is generally around 12 months [240,241]. Overall, the limited available evidence suggests that chemotherapy may retain good efficacy in post-transplant CRC patients.

For targeted therapies, anti-VEGF tyrosine kinase inhibitors are commonly used in patients with recurrent HCCs post-LT. We reported a cohort of 34 such patients receiving sorafenib who achieved a median OS of 14 months [242]. In another retrospective cohort with 45 patients receiving lenvatinib, an ORR of 20%, PFS of 7.6 months and OS of 14.5 months was reported [243]. Retrospective case series on the sequential use of regorafenib after sorafenib in patients with HCC recurrence after LT have reported a median OS from the start of sorafenib of 28.8 months and PFS on regorafenib of 5.9 months [244,245]. These results are broadly in line with the results of registration trials, demonstrating that anti-VEGF TKIs may have preserved efficacy even in post-LT patients [246,247,248]. Lastly, the efficacy of cetuximab was reported in 17 post-renal transplant patients with cSCCs, though efficacy outcomes are difficult to interpret due to a large proportion being treated in the adjuvant setting [249].

## 5. Implications on Expansion of Transplant Indications and Future Directions

Advancements in systemic therapeutics have driven two trends in transplant oncology: firstly, more locally advanced tumors can be downstaged to permit a curative transplant. Take the example of advanced HCC: ICI combinations have a higher ORR compared to TKIs, the previous standard of care. This allows for more patients originally beyond Milan or UCSF criteria to shrink their tumors and be eligible for curative LT afterwards. Secondly, more therapy options after transplantation may be available if the patient relapses afterwards or has a de novo cancer, prolonging survival and even inducing long-term disease control in a minority of patients. Recently, LT has also been used to ‘rescue’ insufficiently effective systemic therapy, being used as a curative treatment in patients with CRCLM in whom systemic therapy was insufficient to downstage the tumor to permit resection [250]. All of these trends may drive efforts to expand indications for transplantation in oncology patients. However, it is also important to keep in mind that anti-tumor immunity may be significantly disrupted after transplantation and immunosuppression, driving the relapse of tumors and limiting the efficacy of systemic therapies. To optimize the outcomes of transplants and minimize waste of finite grafts, further studies should be made to optimize anti-tumor immunity in transplant recipients. Two of the principal difficulties are that certain key factors promoting graft tolerance may also promote tumor immune escape, and that generally the quantity and quality of clinical data is limited.

To target these issues, future research should be focused on the following aspects. Clinically, strategies to optimize ISTs should be developed. These include exploring alternatives to agents such as ATG and seeking novel approaches, such as regulatory T-cell transfer, to minimize lifelong conventional immunosuppression and thus impairment of anti-tumor immunity [251]. Precision immunophenotyping to design targeted immunosuppression strategies in patients with different risks and types of rejection should also be done to reduce general overtreatment or broad suppression of oncologically useful immune cell repertoires [252]. This approach would also be potentially extremely useful in selecting patients for ICI therapies by allowing for the prediction of rejection risks from immune checkpoint blockade. In terms of clinical data, the reporting of treatment efficacy, stratified by graft and tumor types, of different types of anti-cancer therapy in SOT recipients should be done. Next-generation immunotherapies to reinvigorate anti-tumor response while posing lower risks of triggering rejections than ICIs, such as the oncolytic immunotherapy vusolimogene oderparepvec which is currently under trial in SOT recipients with advanced cutaneous malignancies, should also be explored [253].

Translationally, further characterization of the immune profile in patients with SOT and the impact of different factors such as the graft microenvironment and ISTs on immune cells should be done. Ideally, this should be performed for SOT recipients with tumors and longitudinally tracked as they receive systemic therapies to understand the unique dysfunctional states of anti-tumor immune cells at baseline and in response to stimuli. To hone strategies for patient selection and rejection prevention, changes in immune cell subsets and functions, as well as biomarkers such as immune checkpoints, and cytokine profiles in both peripheral blood and the graft organ in patients with SOT given ICIs should be delineated, both for those who developed rejection and those who did not. Lastly but most importantly, given the scarcity of data, global collaboration is required to build international cohorts and prospective studies to improve the outcomes in these patients and allow more patients to benefit from curative transplantation.

## 6. Conclusions

Anti-tumor immunity may be impaired by immune cell dysfunction and unique graft microenvironmental factors. All systemic treatment modalities employ immune-mediated mechanisms to kill cancer cells. ICIs in SOT patients showed relatively preserved efficacy in cSCCs but not in other tumor types, while data for other systemic therapy modalities are scarce. Further clinical and translational research is required to improve the outcomes of SOT recipients with cancer and pave the way towards increasing the number of patients that would benefit from transplantation.

## Data Availability

No new data were created or analyzed in this study. Data sharing is not applicable to this article.
